# Supplementary material for: Evaluating the survival and removal of Escherichia coli from surfaces made with traditional and sustainable cement-based materials in field-relevant conditions
Source: Appl Environ Microbiol. 2025 Mar 10;91(4):e02131-24. doi: 10.1128/aem.02131-24 (PMC12016513; doi:10.1128/aem.02131-24)
Supplement: Supplemental material — Figure S1, Tables S1 and S2, Bangladeshi cement mix design, and cement sample mix protocol. [file aem.02131-24-s0001.pdf]

Supplemental Material

Evaluating the Survival and Removal of *Escherichia coli* from Surfaces Made with Traditional and Sustainable Cement-Based Materials in Field-Relevant Conditions

Claire E. Anderson [a+*], Jason Hernandez [a+], Suhi Hanif [b], Lauren Owens[c], Yoshika Crider[d], Sarah L. Billington[a], Michael Lepech [a], Alexandria B. Boehm [a], Jade Benjamin-Chung [b,e]

[a] Department of Civil and Environmental Engineering, Stanford University, Stanford, CA, 94305
[b] Department of Epidemiology and Population Health, Stanford University, Stanford, CA, 94305
[c] Department of Computer Science, Stanford University, Stanford, CA, 94305
[d] King Center on Global Development, Stanford University, Stanford, CA, 94305
[e] Chan Zuckerberg Biohub, San Francisco, CA, 94158
+ Denotes equal contribution
*Author to whom correspondence should be addressed: Claire E. Anderson, claire34@stanford.edu

The supplemental material contains the following information, as referenced in the main article:

Figure S1: Concrete tile and removal activity materials………………………………………………………… S2

Table S1: Mix design proportions…………………………………………………………………………………… S3

Table S2: Fine aggregate mix properties………………………………………………………………………… S4

Bangladeshi cement mix design………………………………………………………………………………………. S5

Cement samples mix protocol……………………………………………………………………………………… S7

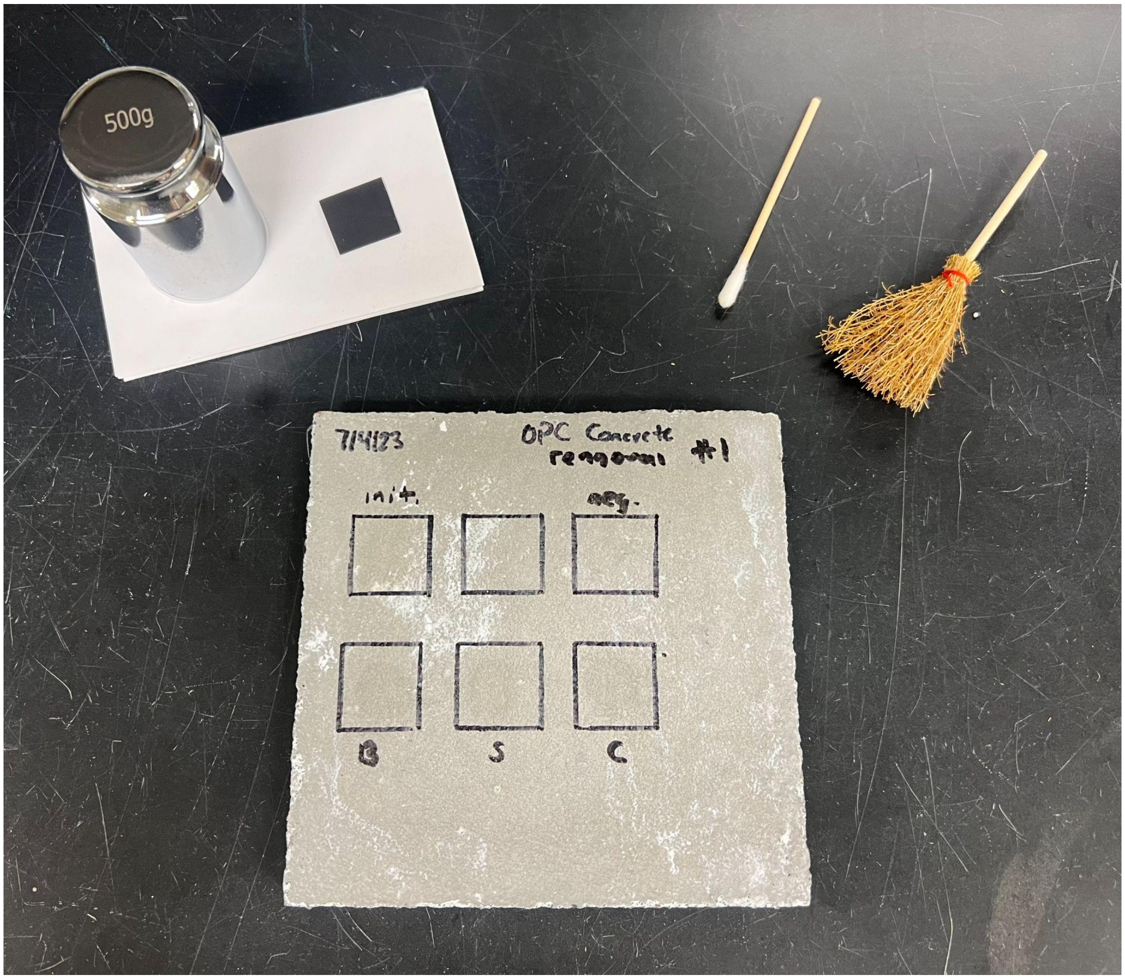

Figure S1: Concrete tile and removal activity materials. On the white paper in the upper left of the image, there is the rubber square and 500 g weight used to mimic walking, the upper middle shows the cotton swab used to mimic mopping, and the upper right shows the miniature straw broom used to mimic sweeping. The tile is in the lower center, with the 2 cm squares for each sample outlined.

Table S1: Mix design proportions for Ordinary Portland Cement (OPC) concrete, OPC mortar, OPC fly ash mortar, and neat cement finishes. OPC fly ash mortar is a mortar mix with a 25% fly ash substitute.

**Mix Design**	**Water-to-Cementitious Material Ratio**	**Cement (kg/m^3)**	**Water (kg/m^3)**	**Fine Aggregate (kg/m^3)**	**Coarse Aggregate (kg/m^3)**	**Supplementary Cementitious Materials (kg/m^3)**
OPC Concrete	0.466	509	245	738	750	–
OPC Mortar	0.466	630	294	1200	–	–
Neat Cement Finish	0.466	1276	595	–	–	–
OPC Fly Ash Mortar	0.466	473	294	1200	–	158
Neat Cement Finish with Fly Ash	0.466	957	595	–	–	319

Table S2: Fine aggregate mix properties. American Standard Test Sieve Series (A.S.T.M) and British Standard Sieve (B.S.S.) sieve sizes and percent passing aggregate are compared.

Study Sample Gradation				
**Sieve Number (A.S.T.M.)**	**Sieve Size (mm)**	**Percent Passing (%)**	**Physical Properties**	
4	4.75	100	Organic Impurities	Satisfactory
8	2.36	96	Sand Equivalent	78
16	1.18	80	Durability	76
30	0.6	37	Specific Gravity	2.56
50	0.3	10	Absorption	0.8%
100	0.15	2		
200	0.075	1		

Bangladesh Gradation		
**Sieve Number (B.S.S.)**	**Sieve Size (mm)**	**Percent Passing (%)**
7	2.36	90 - 100
14	1.18	75 - 90
25	0.6	45 - 75
52	0.3	30 - 50
100	0.15	0 - 5

Bangladeshi cement mix design

A design plan was provided by Village Education Resource Center (VERC), a non-governmental (NGO) in Bangladesh for the Cement flooRs AnD chiLd hEalth (CRADLE, NCT05372068) trial. Relevant portions of the design plan which were mimicked in the mix design and fabrication of the cement tiles used in this study are included.

Cement Plastering with neat cement finishing

The top of an installed brick layer will be covered by 12 mm thickness cement plastering with net cement finishing. This work consists of the application of cement plaster to brick soling surfaces shown on the Drawings or ordered by the Engineer. Cement mortar for plastering shall conform to 12 mm thick (1:4) with neat cement finishing of the floor. The surfaces to be plastered shall be cleaned of all dust and mud by brushing and scraping. All loose flakes of brick, mortar, or stone shall be removed with a trowel, or a hammer and chisel. Bricks to be plastered shall be thoroughly wetted and maintained wet for at least 24 hours before any plaster is placed on them. The average thickness of the final layer of plaster shall be not less than the specified thickness by more than 03 mm. In local depressions where the masonry surface requires more than 20 mm of plaster, an initial leveling layer of plaster shall be applied and allowed to set for 24 hours prior to applying the final surface. The leveling layer shall be left rough and grooved by trowel cuts to ensure a good bond with the final layer. The mortar for the final layer of plaster shall be applied in a fairly wet condition as a rough layer of approximately the required thickness. The evenness of the layer shall be continuously checked using a wooden straight edge. Mortar stricken off the plastered surface shall not be reused. Such work using reused plaster shall be rejected and redone to the satisfaction of the Engineer at the contractor's expense. When the plaster has achieved its initial set and feels firm under the fingertips, the surface shall be smoothed using a wooden float. Where required by the Engineer, the plaster surface shall be finished using a steel float or trowel and a light application of water as required to seal the surface. A full wall or ceiling shall be completed in one day. All plastered surfaces shall be absolutely plain and free of joint patches or laps. Corners and edges shall be finished as shown on the Drawings or as otherwise directed by the Engineer.

Note: A predetermined number of homes will have floors installed using a 25% fly ash of Portland cement for both the 12 mm thick cement plastering and the neat cement finish. The steps for these floors will be the same as the normal floors.

Curing: The surfaces shall then be thoroughly wetted, and kept wet for at least seven days. During this period it shall be suitably protected from all damages.

Testing: Compressive strength testing will be done every 10th floor that is installed for each mix (100% OPC and 25% fly ash). Three 51mm x 51mm cube samples will be fabricated from a single 3-piece mold using material straight from the mixer. The samples will be removed from the mold after 24 hours and wet-cured for 7 days in a lime water bath. Each sample will be labeled with the mix type (OPC or FA), date poured (mm/dd/yyyy), and an identifier for which household it is from. The samples will be sent to the Blume Earthquake Engineering Center at Stanford University (439 Panama Mall, Stanford, CA 94305).

Materials Specifications

Portland Cement: All cements shall be ordinary Portland cement conforming to the requirement of ASTM Type 1 or BS12. The compressive strength of a standard cube shall not be less than for 03 days, 07 days and 28 days are 13 N/mm^2, 20 N/mm^2 and 28 N/mm^2 respectively. The tensile strength of standard cubes is 1 N/mm^2, 2 N/mm^2 and 2.5 N/mm^2 respectively. Initial setting time of cement shall not be more than

30 minutes and final setting time not more than 08 hours. The unit weight of cement shall be 14.16 kN/m^3. The cement when tested for soundness shall not have an expansion of more than 10 mm. Cement shall be delivered at the work site in sound and properly sealed bags, each plainly marked with the manufacturer's name. The cement shall be protected from the weather by covering it during transit. The weight of individual bags containing cement shall be 50 kg. The use of cement reclaimed from discarded or used bags will not be permitted. All cements shall have the written approval of the Engineer prior to being used in the works. The contractor is to produce samples of cement from their stock at the site for physical and chemical tests as required under the Contract or as on demand of the Engineer. The contractor shall undertake at their own cost necessary tests for cement as and when asked by the Engineer.

Water: The water used for the preparation of concrete or mortar shall be clean, potable, free from objectionable quantities of silt, organic matter, alkali, sulfates, other salts and other impurities, and will be subject to approval by the Engineer.

Coarse Aggregate: Coarse aggregate for concrete shall consist of either (i) stone chips or (ii) brick chips. The brick chips shall be made from 1st class picked over burnt (jhama but not porous) bricks, having a minimum compressive strength of 175 kg/cm for individual bricks and an average of 210 kg/cm^2, as may be specified on the drawings and in the Schedule of Works. Chips shall be graded from 05 mm to 20 mm with 33% passing 10 mm sieve unless otherwise determined from the laboratory trial mixes or as directed by the Engineer. All coarse aggregates shall be cleaned and made free from dust and other impurities by screening and washing in clean water immediately before use.

Fine Aggregate: Fine aggregates for concrete shall consist of a blend of coarse sand (Sylhet/Domar with a FM of minimum 2.5) and medium sand having a minimum FM of 1.80 in accordance with the gradation in Table S2. Fine aggregates shall be free from clay particles and other deleterious materials. Organic materials content shall not exceed 05% and silt and other fine materials content shall not exceed 06%. All fine aggregates shall be screened and washed in clean water immediately before use. Fly Ash will meet classification requirements for Class F Fly Ash per ASTM C618. The amount of fly ash used is measured at a 1:1 replacement of cement at 25%. The replacement amount is the upper limit allowed per ACI 232.2R. Fly ash will be used in both the 12 mm cement plaster and the neat cement finish at a 1:1 replacement of cement at 25%.

Cement samples mix protocol

The mixing procedure for the neat cement finish and mortar mixes was adopted from *ASTM C305 Standard Practice for Mechanical Mixing of Hydraulic Cement Pastes and Mortars of Plastic Consistency*. The mixing procedure for the concrete mix was adapted from A*STM C192 Standard Practice for Making and Curing Concrete Test Specimens in the Laboratory*.

Procedure for mixing paste

1. Place all mixing water in the bowl
2. Add cement and allow 30s for absorption
3. Start mixer and mix at slow speed (140 ± 5r/min) for 30 seconds
4. Stop mixer for 15s and scrape down into the batch any paste
5. Mix at medium speed (285 ± 10r/min) for 60 seconds

Procedure for mixing mortar

1. Place water into bowl
2. Add cement to the water and mix at slow speed (140 ± 5r/min) for 30 seconds
3. Add sand while mixing at slow speed over 30 seconds
4. Stop mixer and mix at medium speed (285 ± 10r/min) for 60 seconds
5. Stop mixer and let mortar sit for 90 seconds
6. During first 15 seconds, quickly scrape any mortar that was collected on the side
7. Mix at medium speed (285 ± 10r/min) for 60 seconds

Procedure for mixing concrete

1. Add aggregates to mixer with half of the water and mix for 1 minute
2. Stop mixer and add cement material to mixer and mix for 30 seconds
3. Add rest of water over one minute
4. Mix all the ingredients for 3 minutes followed by 3 minutes of rest
5. Mix a final time for 2 minutes
